# Effect of hay supplementation timing on rumen microbiota in suckling calves

**DOI:** 10.1002/mbo3.430

**Published:** 2017-12-27

**Authors:** Xueyan Lin, jian Wang, Qiuling Hou, Yun Wang, Zhiyong Hu, Kerong Shi, Zhengui Yan, Zhonghua Wang

**Affiliations:** ^1^ College of Animal Science Shandong Agricultural University Taian Shandong China

**Keywords:** calves, hay supplementation timing, rumen fermentation, rumen microbiota

## Abstract

An animal feeding trial was conducted on 18 seven‐day‐old Holstein dairy bull calves weighing 42 ± 3 kg each. Calves were randomly assigned into three groups (*n* = 6 each). The dietary treatments were as follows: (1) milk and starter for the control group (MS), (2) supplementation of oat hay from week 2 on the basis of milk and starter (MSO2), and (3) supplementation of oat hay from week 6 on the basis of milk and starter (MSO6). All animals were fed starter and oat hay ad libitum. The major phyla in the different groups of rumen fluid included Firmicutes, Actinobacteria, Bacteroidetes, Proteobacteria, and Euryarchaeota. The major genera were identified, and major genera proportions in the three groups were as follows: *Methanobrevibacter* (Euryarchaeota), 2.1%, 1.7%, and 2.1%; *Olsenella* (Actinobacteria), 23.9%, 17.7%, and 12.8%; *Prevotella* (Bacteroidetes), 10.5%, 16.5%, and 19.2%; *Dialister* (Firmicutes), 3.3%, 4.1%, and 2.8%; *Succiniclasticum* (Firmicutes), 3.8%, 4.7%, and 9.2%; and *Sharpea* (Firmicutes), 0.4%, 2.5%, and 0.2%, respectively. There were no significant differences in the various phyla among the three groups (*p* > .05). The results showed that calves hay supplementation time did not affect the diversity of the rumen microbiota in the suckling calves. However, the hay supplementation altered the proportion of the various microbial populations, supplementation of oat hay from week 2 on the basis of milk and starter could improve calves rumen pH.

## 1 Introduction

The digestive tract of all animals is colonized by a large number of microbes, and these microbes are more abundant in the rumen. These microbial populations (microbiota) not only undergo material and information exchange with the body, but also participate in the metabolism of the host and maintain the body's homeostasis and rumen's normal digestion function. The composition of the entire microbiota and the population levels in the rumen are affected by various factors, among which diet is the most important. Solid feed effect on fibrolytic and methanogenic microorganisms number in the gastrointestinal tract of calves (Guzman et al., 2016). A consensus on the breeding of calves is to encourage the use of digestible starter in the suckling period. However, whether to supplement the suckling calves with hay remains controversial. For example, most producers in the United States choose to supplement hay for weaning calves mainly fed on milk and starter; this is because early feeding of hay will partially replace the feeding of starter and thus affects the rumen development of the calves (Drackley, 2008). In the United Kingdom, hay is mainly supplemented for calves at 2–3 weeks of age; the calves are also fed on starter and milk to promote rumination, increase rumen pH, and benefit rumen health and development (Castells, Bach, & Terré, 2012; Coverdale, Tyler, Quigley, & Brumm, 2004; Khan, Weary, & Von Keyserlingk, 2011). The above two feeding systems seem to be contradictory.

So we have researched on the calves hay supplementation time effect on rumen development and production performance. Our laboratory have got the results that the production performance and rumen development were better in the calves with hay supplementation from the second week, but the mechanism of it was not clear. So here, we examined the microbiology ecology change and microbiology fermentation on the condition that different calves supplementation of oat hay time, which hope to get the microbiology actin in it. The study of oat hay supplementation timing in calves explored the effect of hay supplementation in different periods on the rumen microbiota in calves from a microbial perspective.

## 2 Experimental Procedures

### 2.1 Experimental animals and design

We selected 18 seven‐day‐old Holstein dairy bull calves in similar body condition, weighing 42 ± 3 kg each. Calves were randomly assigned into three groups, with oat hay as the supplement: Group 1: milk + calf starter (MS); Group 2: milk + calf starter + oat hay, with oat hay supplemented from week 2 (MSO2); and Group 3: milk + calf starter + oat hay, with oat hay supplemented from week 6 (MSO6). Calves were provided with clean water, commercial starter, and imported oat hay ad libitum. The calves were weaned at 9 weeks of age, feeding trial then ended.

### 2.2 Feeding management

Calves were fed in separate pens on a calf island created with sand. Milk was produced on the aote dairy farm in Qingdao, and fed twice a day (0500 and 1700 hr). The dosage of milk was 4 L/calf per day, 2 L each time, until sudden weaning. Calf starter was provided twice a day (0700 and 1800 hr), and the dosage of starter was determined according to feed intake in the previous 2 days. Oat hay was supplemented according to the experimental design, and the dosage was determined based on the feed intake in the previous 2 days. The feed intake and health condition of the calves were checked regularly in the morning, at noon, and during the night. The calf island was cleaned at regular times once a day and disinfected once a week.

### 2.3 Dietary composition and nutrient levels

#### 2.3.1 Commercial calf starter

The commercial calf starter comprised corn, soybean meal, cottonseed meal, corn germ meal, corn distillers dried grains, wheat bran, rock flour, calcium hydrogen phosphate, sodium chloride, vitamins and vitamers, enzymic preparations, and antioxidants. The nutritional ingredients of the starter are shown in Table 1.

**Table 1:** Nutritional ingredients of calf starter feed and oat hay (dry matter basis)

Ingredients	Calf starter	Oat hay
Dry matter (%)	88.74	90.32
Crude protein (%)	23.14	5.82
Ether extract (%)	3.68	1.58
Crude ash (%)	7.89	6.79
Neutral detergent fiber (%)	23.37	61.17
Acid detergent fiber (%)	6.78	35.73
NE_L_ (Mcal/kg)	1.76	1.14

NE_L_, calculated net energy for lactation.

#### 2.3.2 Oat hay

The hay was an imported high‐quality oat hay. The nutritional ingredients of the hay are shown in Table 1.

### 2.4 Sample collection and treatment

#### 2.4.1 Rumen fluid sampling and analysis

Rumen fluid samples were collected from the calves at 63 days of age using a rumen fluid collector. Sampling was conducted within 2–3 hr after calves were fed with the starter. Fluid samples were dispensed into two 50 ml centrifuge tubes and immediately frozen in liquid nitrogen for storage. The samples were delivered to the laboratory and slowly thawed at 4°C. Total DNA was extracted from the rumen fluid using the Stool DNA Isolation Kit (Tiangen, Beijing, China). The DNA samples were sent to RiboBio (Guangzhou, China) for V4 region of the 16s rDNA gene sequencing.

### 2.5 Data analysis

Raw data were sorted using Excel (Microsoft Corp., Redmond, WA, USA). Statistical analysis was performed using the ANOVA module in SAS 8.0 (SAS Institute Inc., Cary, NC, USA). Differences in the means were tested using Duncan's multiple comparison test. A *p* < .05 indicates a significant difference, and a *p* < .01 indicates a highly significant difference in sequencing of the V4 region of the 16s rDNA gene. If the two paired end reads overlapped, the consensus sequence was generated by FLASH. Tags were redundant with Mothur (version 1.27.0). Then, we selected unique tag. Unique tag was preclustered by means of SLP, the tags were then clustered into OTU with a 97% threshold by using UCLUST (Edgar, 2010; Huse & Mark Welch, 2010).

## 3 Results

### 3.1 Sequencing and classification

A total of 1,583,597 reads were obtained from the sequencing results after quality control. There were 528,692, 535,116, and 519,789 reads in the MS, MSO2, and MSO6 groups, respectively, with an average number of 93,153 reads per sample. Operational taxonomic units (OTUs) were obtained at 97% similarity. At an allowable sequencing error rate of 0.1%, the average sequencing quality score Q30 was 88.06% > 62.65%. This result indicates that the sequencing quality was good and thus was adequate for the subsequent data analysis.

### 3.2 Alpha diversity analysis

**Figure 1:**
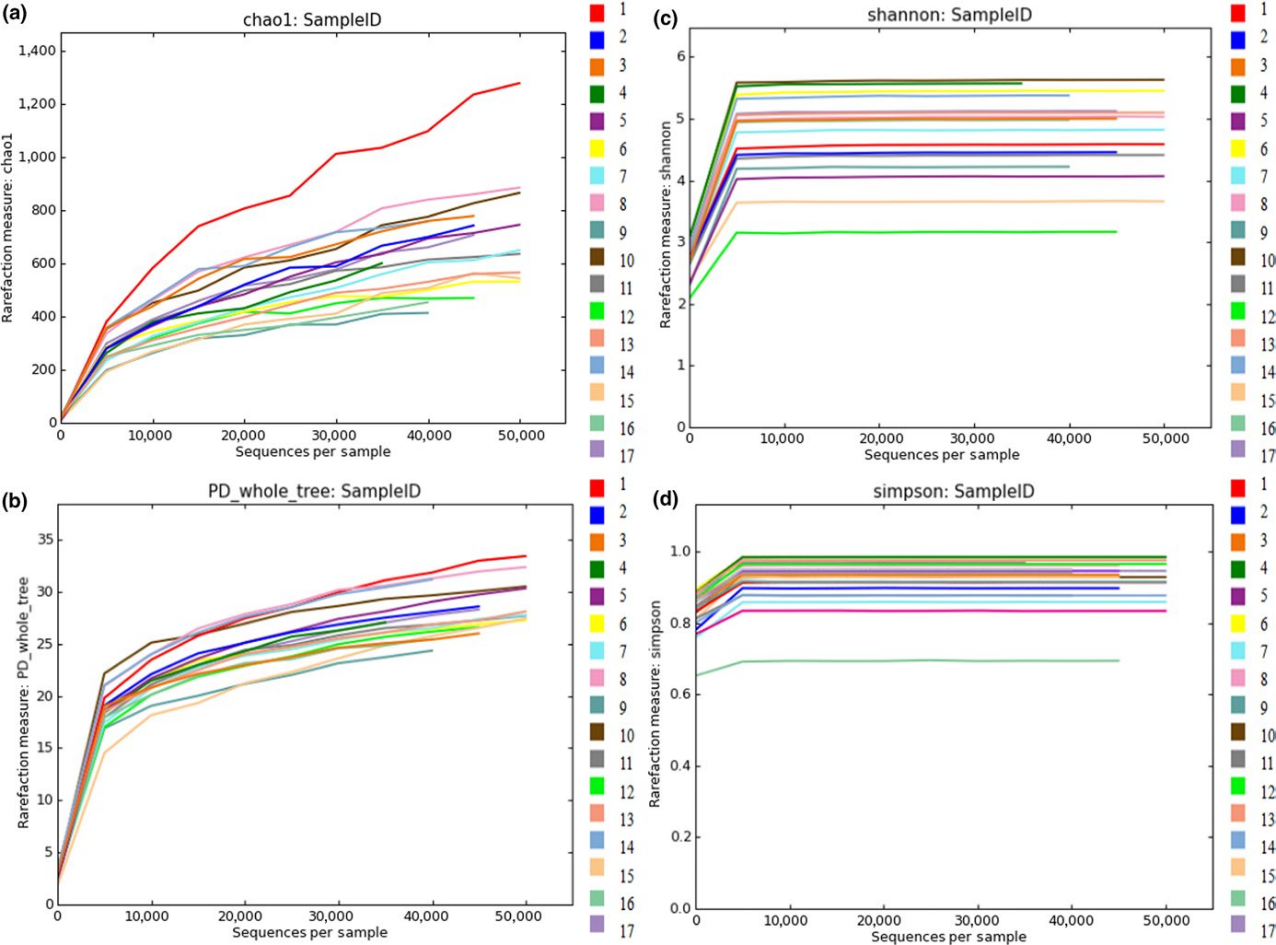
Refraction curves of the (a) Chao index, (b) PD_whole_tree index, (c) Shannon index, and (d) Simpson index

The alpha diversity was evaluated using Chao, PD_whole_tree, Shannon, and Simpson indices (Figure 1). The four groups of rarefaction curves tended to flatten out or reach a plateau, indicating that the sequencing depth generally covered all species.

In Table 2, the chao1 index estimates the number of OTUs in the microbial community. There were significant differences in the chao1 index between the groups (*p* < .05), indicating not similar species richness in rumen fluid samples. The PD_whole_tree index showed no significant difference among the groups (*p* > .05), indicating similar species origin in the rumen fluid samples. The Shannon diversity index showed no significant difference among the groups (*p* > .05), indicating similar species diversity in the rumen fluid samples. The Simpson diversity index showed a not significant difference among the groups (*p* > .05), indicating a similar community richness in the rumen fluid samples. None of these indices were significantly different among the dietary groups. This result may indicate that the community diversity of the microbiota in the rumen fluid was similar between the various groups of calves and did not change with diet.

**Table 2:** Comparison of the four alpha diversity indices between the dietary groups

Index	Dietary treatment			SEM	*p*
	MS	MSO2	MSO6		
Chao	904	690	633	247.2	.17
PD_whole_tree	30.1	28.6	28.6	2.94	.67
Shannon	4.7	4.9	4.6	0.68	.69
Simpson	0.91	0.92	0.88	0.067	.65

### 3.3 Beta diversity

**Figure 2:**
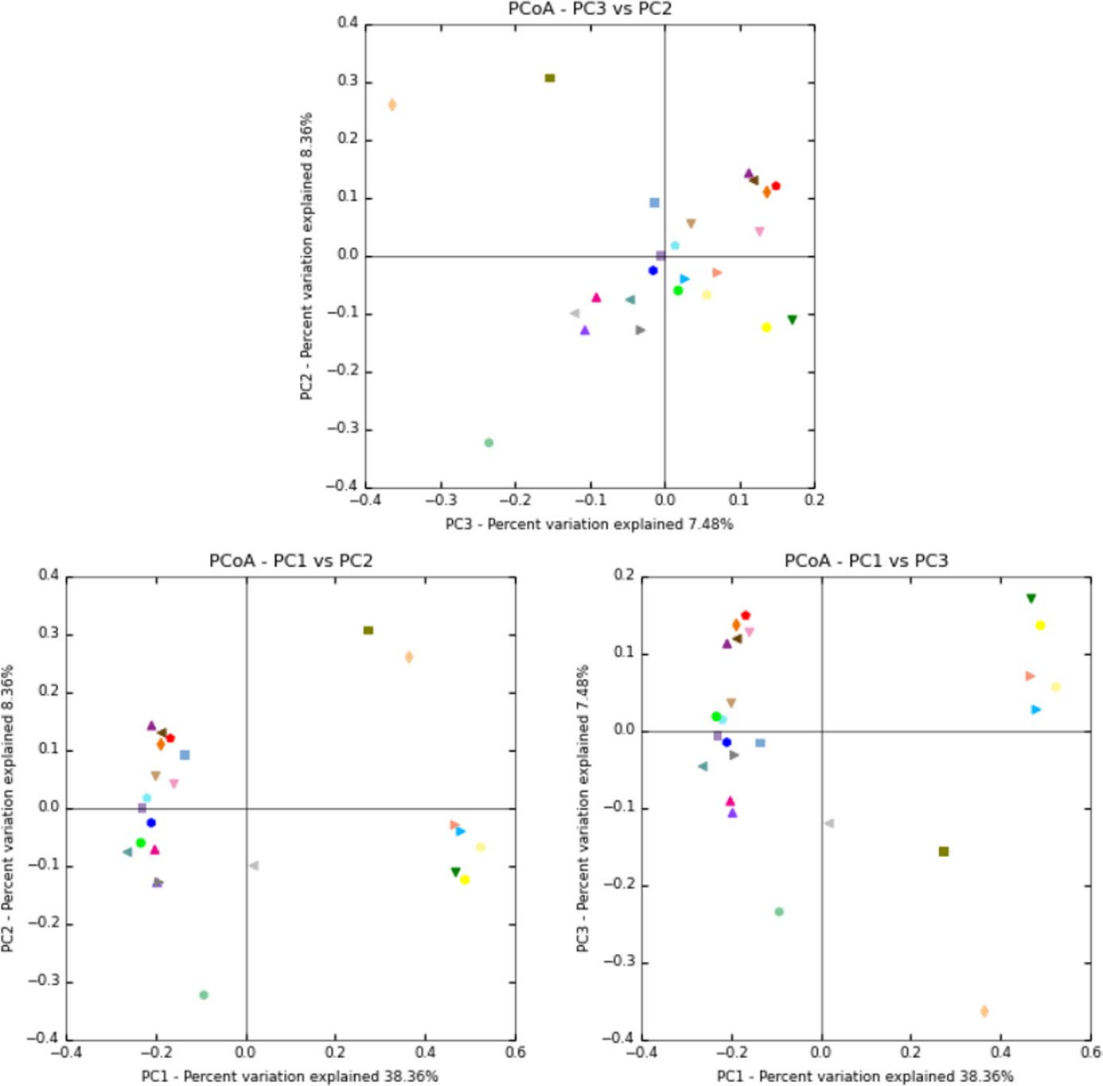
PCoA

We used a beta diversity analysis to examine the difference in the microbial community structure among the samples. The closer the beta diversity value is to 0, the smaller the difference is in species diversity between the two samples. Table 3 shows beta diversity values between the corresponding samples in the horizontal and vertical directions. The larger the value is, the greater the difference between the two samples, and vice versa (Table 3; Figure 2). The results suggested that there were bigger difference between sample 12 and 15, 5, 10, 2, 1, 11, 13, between sample 9 and 2, 4, 11, 15, 16. Note: treatments MS,c1,1–6; MSO2,c2,7–12; MSO6,c3,13–17.

**Table 3:** Beta diversity in rumen fluid of calves

Calf	15	1	5	3	17	10	8	14	6	11	13	2	9	16	12	7	4
15	0	0.4	0.3	0.5	0.7	0.5	0.4	0.8	0.6	0.2	0.6	0.2	1.1	0.4	1.2	0.4	0.5
1	0.4	0	0.3	0.4	0.4	0.4	0.4	0.7	0.4	0.3	0.4	0.2	0.9	0.3	1.0	0.2	0.4
5	0.3	0.3	0	0.3	0.4	0.4	0.2	0.6	0.4	0.2	0.4	0.3	0.9	0.4	1.0	0.3	0.4
3	0.5	0.4	0.3	0	0.2	0.3	0.2	0.4	0.3	0.4	0.3	0.5	0.8	0.4	0.8	0.4	0.3
17	0.7	0.4	0.4	0.2	0	0.3	0.3	0.4	0.3	0.5	0.3	0.5	0.8	0.4	0.8	0.3	0.4
10	0.5	0.4	0.4	0.3	0.3	0	0.3	0.5	0.2	0.3	0.2	0.4	0.9	0.2	1.0	0.3	0.2
8	0.4	0.4	0.2	0.2	0.3	0.3	0	0.5	0.3	0.3	0.3	0.4	0.9	0.4	0.9	0.3	0.3
14	0.8	0.7	0.6	0.4	0.4	0.5	0.5	0	0.5	0.7	0.4	0.7	0.7	0.6	0.8	0.5	0.6
6	0.6	0.4	0.4	0.3	0.3	0.2	0.3	0.5	0	0.5	0.2	0.5	0.9	0.4	0.9	0.4	0.3
11	0.2	0.3	0.2	0.4	0.5	0.3	0.3	0.7	0.5	0	0.5	0.2	1.0	0.3	1.1	0.4	0.4
13	0.6	0.4	0.4	0.3	0.3	0.2	0.3	0.4	0.2	0.5	0	0.5	0.9	0.3	0.9	0.3	0.3
2	0.2	0.2	0.3	0.5	0.5	0.4	0.4	0.7	0.5	0.2	0.5	0	1.0	0.3	1.1	0.3	0.4
9	1.1	0.9	0.9	0.8	0.8	0.9	0.9	0.7	0.9	1.0	0.9	1.0	0	1.0	0.5	0.8	1.0
16	0.4	0.3	0.4	0.4	0.4	0.2	0.4	0.6	0.4	0.3	0.3	0.3	1.0	0	1.0	0.3	0.2
12	1.2	1.0	1.0	0.8	0.8	1.0	0.9	0.8	0.9	1.1	0.9	1.1	0.5	1.0	0	0.9	1.0
7	0.4	0.2	0.3	0.4	0.3	0.3	0.3	0.5	0.4	0.4	0.3	0.3	0.8	0.3	0.9	0	0.4
4	0.5	0.4	0.4	0.3	0.4	0.2	0.3	0.6	0.3	0.4	0.3	0.4	1.0	0.2	1.0	0.4	0

MS, 1–6; MSO2, 7–12; MSO6, 13–17.

### 3.4 Heatmap of OTU richness

**Figure 3:**
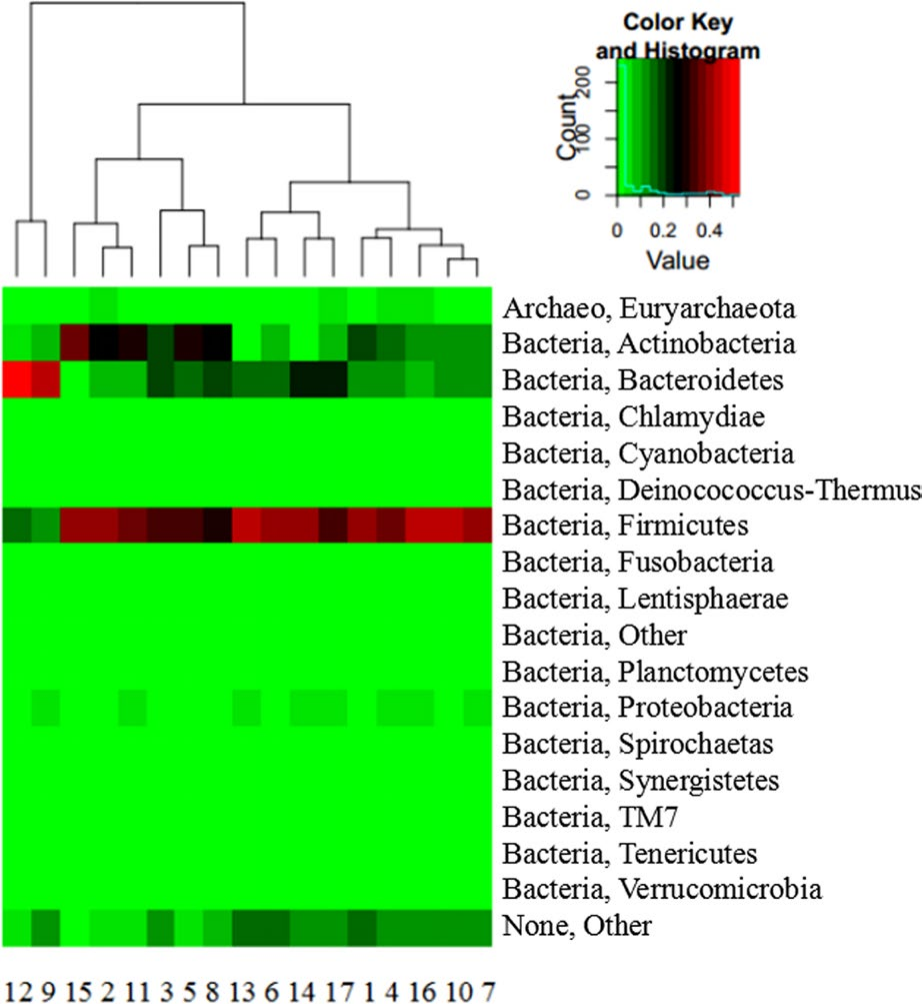
Heatmap showing species richness in the rumen fluid samples of the calves. Note: MS group, 1–6; MSO2 group, 7–12; MSO6 group, 13–17

The information of species composition at the optimal classification level was clustered according to the distance between the samples and the species. Horizontal (sample clustering) and vertical clustering (species classification) was achieved on the basis of species richness levels by unsupervised hierarchical clustering analysis. This procedure examines the degree of aggregation of samples and detects similar samples and species; it also reflects the richness differences in the samples. The major phyla showing differences between samples included Bacteroidetes, Actinobacteria, and Firmicutes (Figure 3).

### 3.5 Microbiota composition analysis

**Figure 4:**
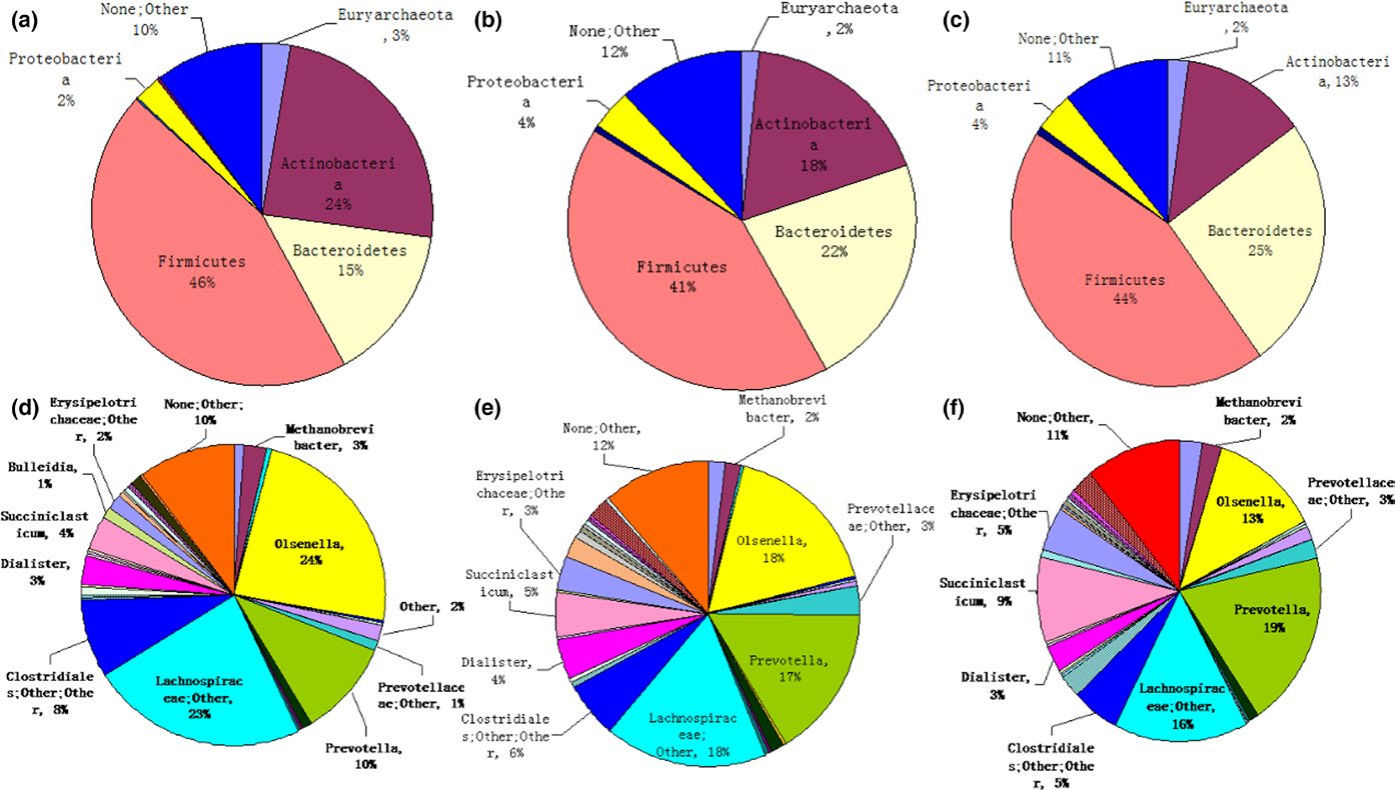
Distribution of the rumen microbiota at the phylum (a–c) and genus (d–f) levels in the MS, MSO2, and MSO6 groups

The OTU sequences were subject to species annotation using the existing 16s database, followed by taxonomic classification into phylum, class, order, family, genus, and species. The number of tags at the different classification levels was counted for each sample. Based on the results, we identified the optimal classification level. Richness analysis was conducted according to the number of tags of the different species on annotations at each classification level. As shown in Figure 4, the annotation of samples indicates the proportion of tags of the specific species in the total tags of the sample. A total of 14 phyla were detected in the rumen fluid of all calves. We compared five major phyla and obtained their proportions in MS, MSO2, and MSO6 groups: Firmicutes, 46%, 41%, and 44%; Bacteroidetes, 15%, 22%, and 25%; Actinobacteria, 24%, 18%, and 13%; Euryarchaeota, 3%, 2%, and 2%; and Proteobacteria, 2%, 4%, and 4%, respectively (Figure 4).

In total, 105 genera were accurately detected in the rumen fluid of all calves. The average proportions of the six genera were greater than 1%, and their proportions in the MS, MSO2, and MSO6 groups were as follows: *Methanobrevibacter* (Euryarchaeota), 2.1%, 1.7%, and 2.1%; *Olsenella* (Actinobacteria), 23.9%, 17.7%, and 12.8%; *Prevotella* (Bacteroidetes), 10.5%, 16.5%, and 19.2%; *Dialister* (Firmicutes), 3.3%, 4.1%, and 2.8%; *Succiniclasticum* (Firmicutes), 3.8%, 4.7%, and 9.2%; and *Sharpea* (Firmicutes), 0.4%, 2.5%, and 0.2%, respectively.

We can see from the results that MSO2 group *Sharpea and Dialister* genera were greater in Firmicutes. MSO6 group *Prevotella* genera were greater in Bacteroidetes.

### 3.6 pH and volatile fatty acid concentration in the rumen fluid

Table 4 shows the effect of timing of hay supplementation on the rumen pH and volatile fatty acid (VFA) concentration in calves. The pH value was the largest in the rumen fluid of the MSO6 group, followed by the MSO2 group; the pH values were lowest in the rumen fluid of the MS group (*p* < .05). However, there were no significant differences among the groups in the concentrations of the VFA, which included acetate, propionate, butyrate, valerate, and caproate (*p* > .05).

**Table 4:** The pH and volatile fatty acid (VFA) concentrations in rumen fluid of calves. Values with dfferent leters indicate signifcant diffrence (p<.05)

Item	Treatment			SEM	*p*
	MS	MSO2	MSO6		
Acetate (mmol/L)	20.62	28.03	22.64	5.946	.11
Propionate (mmol/L)	24.74	30.95	23.94	8.479	.33
Butyrate (mmol/L)	8.62	10.89	9.25	4.686	.75
Valerate (mmol/L)	1.46	1.07	1.21	0.473	.48
Caproate (mmol/L)	2.75	3.81	2.93	1.232	.34
Total VFA (mmol/L)	58.18	74.75	59.97	18.395	.28
pH	5.39^a^	5.69^ab^	5.99^b^	0.361	.012

### 3.7 Species richness analysis

Tables 5 and 6 compare the MS, MSO2, and MSO6 groups at the phylum and genus levels, respectively. A total of 14 phyla and 105 genera were compared among the three groups. At the phylum level, there were no significant differences in the five major phyla among the groups (*p* > .05). At the genus level, differences were obtained among the groups for eight genera that belonged to four phyla, namely Bacteroidetes, Firmicutes, Planctomycetes, and Proteobacteria. The MSO2 and MSO6 groups were significantly lower than the MS group for *Butyricimonas* (Bacteroidetes), *Parabacteroides* (Bacteroidetes), *Porphyromonas* (Bacteroidetes), *Anaerotruncus* (Firmicutes), *Blastopirellula* (Planctomycetes), and *Comamonas* (Proteobacteria). However, the MSO6 group was significantly lower than the other two groups for *Desulfovibrio* (Proteobacteria) (*p* < .05). Additionally, the level of *Succiniclasticum* (Firmicutes) appeared to be higher in the rumen fluid of the MSO2 and MSO6 groups (*p* = .07).

**Table 5:** Comparison of the MS, MSO2, and MSO6 groups at the major phylum levels (100%)

Phylum	Treatment			SEM	*p*
	MS	MSO2	MSO6		
Euryarchaeota	0.027	0.017	0.021	0.0178	.66
Actinobacteria	0.245	0.18	0.129	0.1222	.31
Bacteroidetes	0.147	0.221	0.249	0.1709	.63
Firmicutes	0.448	0.42	0.442	0.1211	.93
Proteobacteria	0.024	0.038	0.039	0.0207	.44

**Table 6:** Comparison of the MS, MSO2, and MSO6 groups at the some genus levels (%)

Phylum	Genus	Treatment			SEM	*p*
		MS	MSO2	MSO6		
Bacteroidetes	*Butyricimonas*	0.048^a^	0.025^ab^	0.011^b^	0.023	.02
	*Parabacteroides*	0.020^a^	0.005^b^	0.003^b^	0.0098	<.01
	*Porphyromonas*	0.023^a^	0.004^b^	0.001^b^	0.0129	<.01
Firmicutes	*Anaerotruncus*	0.007^a^	0.003^b^	0.002^b^	0.0035	.048
	*Succiniclasticum*	3.76^a^	4.73^ab^	9.24^b^	4.439	.07
Planctomycetes	*Blastopirellula*	0.003^a^	0.000^b^	0.000^b^	0.0017	<.01
Proteobacteria	*Comamonas*	0.002^a^	0.000^b^	0.000^b^	0.0012	.02
	*Desulfovibrio*	0.555^a^	0.590^a^	0.109^b^	0.3842	.046

Values with different letters indicate significant difference (*p* < .05).

## 4 Discussion

The rumen of ruminants is colonized by complex, diverse, and nonpathogenic microbes, including anaerobic fungi, rumen bacteria, and rumen protozoa (Wang, Wang, & Li, 2006). The study of rumen microbes can promote a better understanding of the effect of diet on rumen fermentation. Therefore, rumen microbes are closely related to animal growth and development. To adapt to fiber diets, the number of microbial populations capable of fermenting fiber needs to be increased. Complex dietary structure requires a complex rumen microbiota and will give rise to certain dominant populations. In the current study, the dominant populations in the different dietary groups included Firmicutes, Bacteroidetes, Actinobacteria, Euryarchaeota, and Proteobacteria. The major dominant population mainly belonged to Firmicutes (46%, 41%, and 44% in the MS, MSO2, and MSO6 groups, respectively). A dominance by Firmicutes in the rumen of the three groups of calves is related to the physiological characteristics of this phylum. The second dominant population belonged to Bacteroidetes (15%, 22%, 25% in the MS, MSO2, and MSO6 groups, respectively). A few studies have suggested that Bacteroidetes play a role in the normal development of the digestive tract (Thomas, Hehemann, Rebuffet, Czjzek, & Michel, 2011). Additionally, Bacteroidetes are involved in the metabolism of bile acids and toxins or their conversion into mutagenic compounds (Smith, Rocha, & Paster, 2006). The higher values of Bacteroidetes in the hay‐supplemented groups may imply that Bacteroidetes are more favorable for rumen development, that is, rumen growth and volume increasing of the rumen.

MSO2 group *Sharpea and Dialister* genera were greater in Firmicutes. MSO6 group *Prevotella* genera were greater in Bacteroidetes. The bacteria in Firmicutes can help the cell to absorb of glucose, which can increase the weight. The bacteria in Bacteroidetes can work in carbohydrate decomposition. MSO2 group propionate and butyrate were greater than MSO6 group. MSO2 group pH was lower than MSO6 group. Propionate and butyrate can promote the development of rumen papilla.

We found no differences in the various phyla between the dietary groups (*p* > .05). This result indicates a similar distribution of rumen microbiota in these groups, corresponding to the similar VFA concentrations in the rumen fluid of the calves (Table 4). Belanche et al., (2012) have reported the changes in rumen microbiota of animals fed on high‐fiber and high‐starch diets and found that animals fed on fibrous diets had higher levels of fiber‐degrading microbes and higher VFA concentrations in the rumen. This is different from the current result, possibly because the rumen has not fully developed and the microbes could not adapt to dietary changes in calves in the present study. Another reason may be that the intake of fiber was insufficient to cause major changes in the populations of rumen microbes. Nonetheless, the pH conditions in the rumen were consistent. That is, animals fed on fiber diets had higher rumen pH consistent with previous studies. Similarly, a previous study has shown that feeding concentrate diets with high levels of fermentable carbohydrates also reduces the rumen pH (Beharka, Nagaraja, & Morrill, 1998). This can be related to saliva reflux in the oral cavity of the cattle.

## 5 Conclusions

Hay supplementation time did not affect the diversity of the rumen microbiota in the suckling calves with the condition that supplementation of oat hay from week 2 calves or week 6 on the basis of milk and starter. However, it altered the proportion of the various microbial populations, and their structural differences indirectly affected the rumen pH.

## Conflict of Interest

None declared.
